# Our Cases and Literature Review for Presence of Bladder Hernias in the Inguinal Region in Children

**DOI:** 10.3390/diagnostics13091533

**Published:** 2023-04-24

**Authors:** Alparslan Kapisiz, Ramazan Karabulut, Cem Kaya, Sibel Eryilmaz, Zafer Turkyilmaz, Ali Atan, Kaan Sonmez

**Affiliations:** 1 Department of Pediatric Surgery, Faculty of Medicine, Gazi University, 06560 Ankara, Turkey; 2 Department of Urology, Faculty of Medicine, Gazi University, 06560 Ankara, Turkey

**Keywords:** inguinal hernia, literature review, bladder hernia, children

## Abstract

Background: The rate of bladder injury during inguinal hernia repair in children is not well known. However, it is known that bladder injury during childhood inguinal hernia repair places a serious morbidity burden on children. We sought to determine an algorithm to avoid accidental bladder injuries. Methods: Reports that included pediatric patients with inguinal hernias containing the bladder were searched. Keywords and mesh term searches were conducted in the MEDLINE, Scopus, and Web of Science databases. We reviewed our clinical records and found that two patients had inguinal hernias containing the bladder. Results: Nineteen articles reporting on 26 patients diagnosed with the presence of the bladder within the inguinal canal from 1962 to 2021 were included in this article. Our two patients were added to this group. Diagnoses were made incidentally during genitourinary radiological examinations (*n* = 3), intraoperatively during hernia repair (*n* = 7), or due to clinical symptoms and findings (*n* = 18) after standard hernia repair. Bladder augmentation was required for three patients. Conclusions: During the operation, if there is any suspicion regarding the presence of the bladder in the inguinal region, we suggest performing a preoperative cystogram to confirm the position of the bladder and its injury. We recommend that the sac should be opened and the contents inspected before performing transfixion during high ligation of the hernia sac.

## 1. Introduction

Inguinal hernia repair is the most common operation performed by pediatric surgeons. The overall incidence of inguinal hernia in children ranges from 0.8% to 4%. In premature infants, the incidence may rise to 30%, and boys are affected six times more frequently than girls [1,2].

While the bladder is contained in 1% to 4% of inguinal hernias in adults, especially those over the age of 50 years, it is a very rare entity in children. In a single-author inguinal hernia series that included 6361 patients, bladder hernia was reported in only two patients (0.03%) [3,4]. Inguinal hernias of the urinary bladder are subgrouped according to the bladder’s relationship to the peritoneum. Paraperitoneal hernia is the first type, in which the urinary bladder is extraperitoneally located and medially positioned to the herniated peritoneum. This can be seen in either direct or indirect inguinal hernias. Intraperitoneal hernia is another type, in which the herniated urinary bladder is entirely covered with peritoneum. Extraperitoneal hernia is the last type, in which the urinary bladder herniates into the inguinal region while the peritoneum remains in the abdomen. Extraperitoneal bladder hernia is the most common type [5]. Due to the presence of the bladder in the hernia sac, the bladder may be unintentionally injured during hernia repair. The prevalence of bladder injury during inguinal hernia repair has been reported to be between 0.008% and 0.3% [6].

It is known that the medial aspect of the indirect inguinal hernia sac is closer to the internal inguinal ring in infants than in adults. This proximity, in turn, facilitates protrusion of part of the bladder into the inguinal canal in infants [7]. This anatomical position creates a risk of bladder injury during inguinal hernia repair in infancy.

Since the 1960s, some case reports of inguinal bladder hernias in children have been published. In these reports, there are limited data on the development, complications, and management of bladder herniation, especially the important features that the surgeon should be aware of in order to avoid bladder injury. To address these issues carefully, we conducted a systematic review of the literature on patient outcomes and postoperative complications.

## 2. Materials and Methods

**Figure 1:**
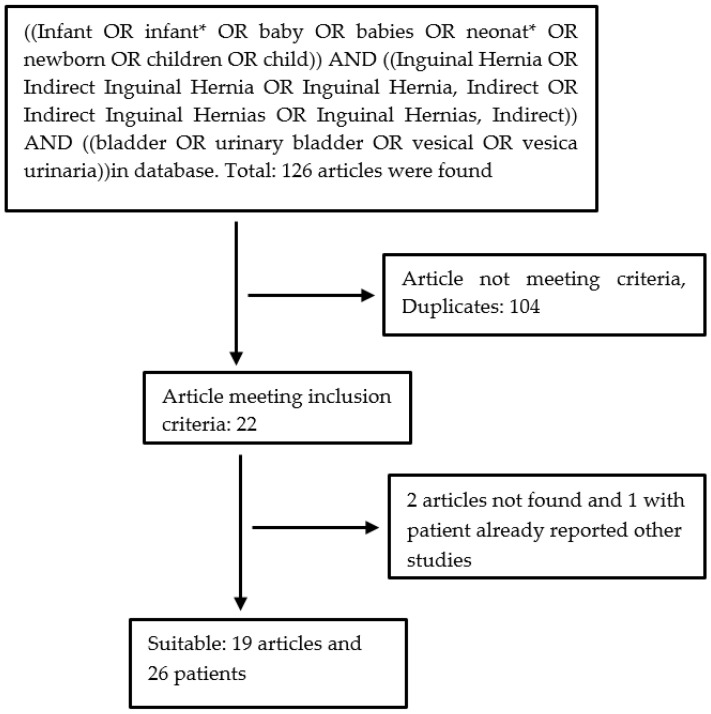
Flow diagram of the literature review.

The PubMed, Web of Science, and Scopus databases were searched electronically on 1 January 2022, utilizing combinations of relevant medical subject heading (MeSH) terms, keywords, and word variants for “((Infant OR infant* OR baby OR babies OR neonat* OR newborn OR children OR child)) AND ((Inguinal Hernia OR Indirect Inguinal Hernia OR Inguinal Hernia, Indirect OR Indirect Inguinal Hernias OR Inguinal Hernias, Indirect)) AND ((bladder OR urinary bladder OR vesical OR vesica urinaria))” (Figure 1). The search and selection criteria were restricted to the English language and full-text publications. Reference lists of relevant articles and reviews were searched for additional reports. The publication span included the past 59 years (1962–2021). A total of 126 articles matching the keywords were found. Excluding duplicates, 22 articles met the inclusion criteria: 2 were not found, and 1 was excluded because it included patients who had been reported in other studies. Nineteen articles were considered for analysis, including a total of 26 patients (Figure 1). The articles were mostly case reports or small retrospective studies.

The inclusion criteria were the diagnosis of an inguinal hernia involving the bladder in patients younger than 18 years of age at the time of diagnosis. If the article included both adult and pediatric patients, only the pediatric part of the data was included in the study. Studies were evaluated according to the following variables: age at the time of diagnosis and clinical features, including symptoms, imaging, hernia side, and surgical details. The same reviewer (RK) extracted the relevant data mentioned above from the included studies. If data belonging to the same patient group were published in more than 1 study, the article containing the most comprehensive information about the population was included in order to not include the same data. The data of two unpublished cases from our department were added to the data set. Selection of these cases was made by reviewing the clinical records of all patients admitted to our department for an inguinal hernia in both elective and urgent settings from January 1990 to December 2021 [5,8,9,10,11,12,13,14,15,16,17,18,19,20,21,22,23,24,25].

Statistical analysis: The results are presented with descriptive statistics. Quantitative variables were summarized as medians and ranges, and qualitative variables were summarized as frequency rates. Statistical analysis was performed using Microsoft Office Excel Professional Plus 2016 program (Microsoft Corporation, Redmond, WA, USA).

## 3. Results

In PubMed, Web of Science, and Scopus, 126 articles matching the keywords were found. Excluding duplicates, 22 articles met the inclusion criteria: two were not found, and one was excluded because it included patients who were reported in other studies. Nineteen articles, including 26 patients, six of whom had a history of prematurity, were included in our study (Figure 1). We added two patients from our clinic to the group. After adding the patients from our center, a total of 28 patients were considered for statistical analysis. 

The median operation age of the patients was 11.8 ± 17.45 months (1–84 months). Twenty-five patients (89.2%) were male, and the sex of one patient was not mentioned. The inguinal hernia was detected on the right side in eleven patients (39.2%), on the left side in six patients (21.4%), and it was bilateral in four patients (14.2%). There were no data with regard to the side in seven patients in the records. In addition, in three of these patients, the patient’s age was not mentioned. Among the patients who had a left-sided inguinal hernia only, for two of the patients who were diagnosed preoperatively, it was not known whether they underwent inguinal hernia repair. Among the patients with bilateral inguinal hernias, the side of the bladder injury recognized intraoperatively or postoperatively was on the right side in two children, and the side was not specified in two children (Table 1). 

The diagnosis was made preoperatively during radiological examination (urography, cystogram, or nuclear study) as genitourinary investigations in three patients (Table 1).

Diagnosis was made in seven patients intraoperatively, two of whom were our patients. In four of these patients, including our patients, bladder injury requiring repair occurred. These injuries were repaired at the same time. In the remaining two patients, the bladder hernia was noticed during the operation. The bladder was reduced without any damage, and Bassini’s procedure was performed. What was performed during the operation for the last patient was not mentioned. All seven patients recovered uneventfully (Table 1).

The diagnosis of bladder hernia was made due to clinical symptoms and findings in 18 patients after hernia repair. The clinical symptoms and findings were urinary leakage or seeping from the incision in six patients, abdominal distension in six patients, anuria in three patients, peritonitis in two patients, and swelling in the inguinal and scrotal region in four patients (Table 1). In the abdominal ultrasound examination, intra-abdominal free fluid was observed postoperatively in two patients [20,23].

It was reported that subtotal or near-total bladder excision was performed in six (21.4%) patients who underwent an operation due to urinary leakage after the first surgery [15,18,20,24,25]. In the postoperative period, bladder augmentation was required due to decreased capacity of the bladder in three patients (10.7%) [13,15,25]. No deaths were reported in the literature during any of these surgical treatments.

## 4. Discussion

Although the rate of preoperative diagnosis of inguinal hernias of the bladder in adults was reported to be less than 10% in Oruç et al.’s study [26], Branchu et al. stated that this rate increased to 60% in the last 10 years [3]. This high rate may be related to the use of more diagnostic studies and more scintigraphy or PET-CT imaging in cancer follow-up, as mentioned by Branchu et al. [3]. According to the screening results in Oruç et al.’s study, 15 cases of late urinary leakage in children and 5 adult patients were diagnosed during the operation, or the perforation was corrected immediately; approximately 79.6% were operated on without a diagnosis of inguinal hernia of the bladder [26].

It is more common in men and on the right side in adult patients. Causes such as urinary outlet obstruction, loss of bladder tone with weakness of the supporting structures, and obesity increase the rate of bladder herniation in adults, which are mostly sliding inguinal direct hernias. Scrotal pressure for voiding in patients over 50 years of age is a specific sign of urinary bladder in the hernia. In contrast, in infants and toddlers, the bladder lies in the abdomen in a superficial position. The sac of indirect inguinal hernias is in close proximity to the bladder medially, or the lateral part of the bladder herniates into the inguinal canal. Transitory extraperitoneal herniation of the bladder is known as “bladder ears”. According to these observations, the bladder may herniate into the internal ring, and the bladder’s grade of protrusion depends on bladder filling [21]. While this close proximity disappears after the sixth month in children, it should be considered that the incidence of hernias increases in premature patients, and hernia repairs are frequently performed before the sixth month [1,13,27].

Even in infants, the bladder has a thinner wall when it is full and extends intra-abdominally to the umbilicus; thus, it can be confused with the hernia sac or hydrocele wall during surgery. Therefore, it is recommended to empty the bladder before inguinal surgery [13]. In fact, in a study by Imamoglu et al. that was not included in this study, factors such as the use of silk sutures during high ligation during the operation and passing from the bladder with a suture due to close proximity were also blamed for cases that presented with pelvic abscesses in the late period after inguinal hernia operations [28].

The rate of bladder involvement during indirect inguinal hernia repair is not well known. Several additional case series describe bladder involvement during pediatric hernia repair. Six cases with recognized bladder involvement and one case involving unrecognized bladder injury were reported by Colodny [22]. In contrast, it was reported to be quite low, at 0.03%, in a large series [4]. In our study, 26 patients were reported, and considering how often inguinal hernia repair is performed all over the world, this low rate will be evident.

Robert Gross said, “There is nothing as interesting as an inguinal hernia”. Indeed, although the operation is considered to be technically straightforward, inguinal hernia repairs can be extremely challenging in small infants. Postoperative complications, which are not uncommon and can be very serious, are almost always caused by errors in technique and are therefore preventable. As a result, this observation is very important and necessitates the utmost attention of surgeons during the operation [29].

The lateral portion of the bladder within the inguinal canal, known as “bladder ears”, was found in 35 of 406 (9%) patients under the age of one year during intravenous pyelography examinations by Allen and Condon. Thirty-three of the 35 patients were less than 6 months of age. They also observed that a bladder ear protruded when the bladder was only partially filled and showed that it disappeared with complete filling of the bladder and with the onset of micturition [7].

While adults usually present with symptoms such as frequency, double voiding, nocturia, urgency, dysuria, and even hematuria, children do not have such symptoms or cannot express themselves due to their young age. Therefore, there is less demand for imaging diagnosis in the preoperative period than in adults [10,30]. Childhood cases and publications on bladder injury relative to adults are limited. In children, most cases of bladder hernias are mainly diagnosed with symptoms such as wound problems due to urinary leakage in the postoperative period, abdominal distention and peritonitis, and conditions that require bladder repair during surgery (79.6%). Papers in which patients had bladder injury and hernia repair performed were included in our study, as these papers were found in the web search using the keywords mentioned above.

The median age at the time of operation in this study was 12.5 months, which is higher than the reported typical age for bladder ears. Bladder hernias and injuries are mostly seen on the right side and in males. This is because inguinal hernias are more common in males. In addition, increased intra-abdominal pressure due to CPAP ventilation and repeated squeezing of the immature intestine are additional predisposing factors and may be the reason why bladder hernias (*n* = 6, 23%) and postoperative recurrences are common in premature patients (33.3%) [21]. Normally, recurrences are seen in 0.8–3.8% of patients after inguinal hernia surgery [31].

Shaw and Santulli reported that out of 2378 consecutive patients undergoing inguinal hernia repair, two infants had been subjected to bladder injury during the operation. One of these two infants had no significant complications, but the other had urinary extravasation, leading to a severe wound infection. These patients were both male and were 3.5 and 9 months old, respectively [14].

Since this anatomical position creates a risk of bladder injury during inguinal hernia repair in infancy, which is a challenging operation, awareness of the presence of the bladder within the inguinal canal and its management are of the utmost importance. If this is not recognized, high ligation of the hernia sac may include the bladder wall. This, in turn, leads to hematuria, possible necrosis of the bladder walls, and extravasation of urine. This situation can be avoided by careful inspection of the neck of the sac at the time of transfixion. When there is any suspicion about this possibility, cystography can demonstrate protrusion of the bladder within the inguinal canal [28].

An excessively fatty and thick structure thought to be a hernia sac within the inguinal canal may herald the presence of the bladder. Unless the physician is aware of this possibility, it may be mistaken for an indirect inguinal sac and result in an inadvertent injury to the bladder. As we experienced in our two patients, an injury of the urinary bladder during hernia repair should not cause serious problems if it is recognized at the time of surgery and repaired appropriately (Table 1).

**Figure 2:**
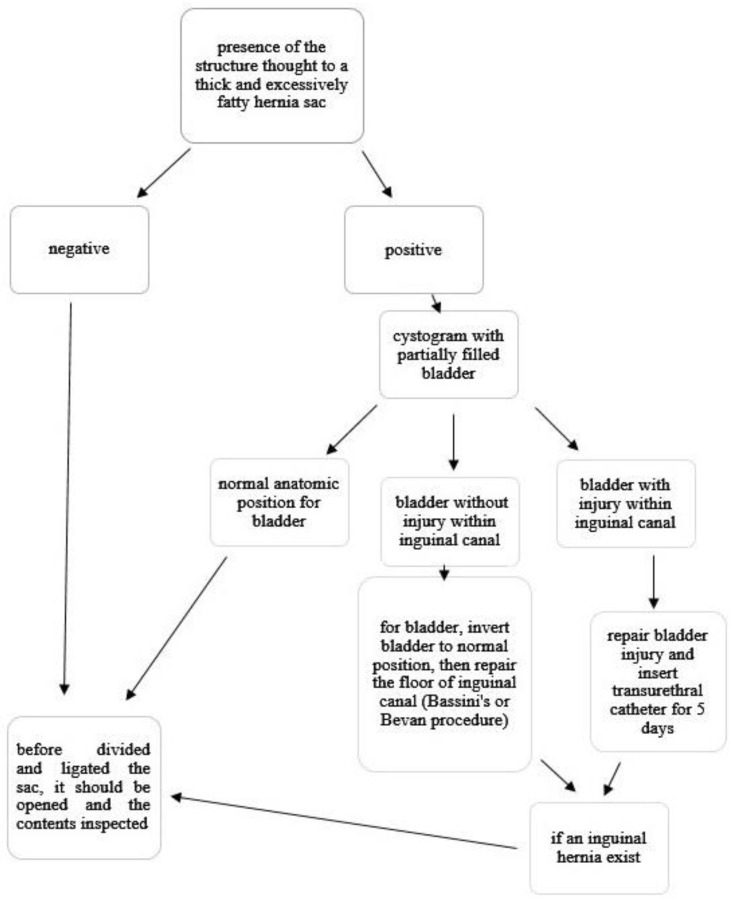
Algorithm.

If the injury is overlooked, it may result in problems, such as a local problem of urinary extravasation, wound infection, urinary ascites, severe azotemia, and potentially death. As has been reported in the literature, the bladder excised partially or nearly totally instead of the hernia sac may lead to a small bladder that requires augmentation (Table 1). We propose an algorithm to avoid all these undesirable complications that we have mentioned (Figure 2).

The limitations of this study are that it is retrospective, we were unable to obtain the details of the operations, there were not always preoperative diagnostic methods, and, especially, there was no postoperative late-period follow-up.

## 5. Conclusions

Awareness of features heralding the presence of the bladder in the inguinal region is crucial to avoid accidental injury of the bladder during inguinal hernia repair in children, especially infants. During the operation, if there is any suspicion regarding this possibility, we suggest performing intraoperative cystography to confirm the position of the bladder and its injury. We recommend that during all inguinal hernia repairs in children, with or without this damage, the sac should be opened and the contents inspected before performing transfixion and excising the distal sac during high ligation of the sac.

## Figures and Tables

**Table 1:** Summary of patients with bladder hernias in the inguinal region (NA = Not Applicable, M = Male, L = Left, E = Elective, U = Urgent).

	Age (Months)	Sex	Side	Surgery Type	Symptoms	Diagnosis	Other Information	Authors
1	12	M	L	NA	Englarged scrotum	Intravenous urography for kidney evaluation	Scrotal cystocele	Foladi et al. [5]
2	1	NA	L	NA	Incidentally	Technetium-99m MAG-3 for evaluation of in utero hydronephrosis	Premature (24 weeks)	Moe et al. [8]
3	6	M	L	E	Bilateral reducible inguinal hernia	Voiding cystourethrogram	Premature (24 weeks), Bassini repair for left-sided hernia	Bhullar et al. [9]
4	24	M	R	E	Inguinoscrotal swelling	Intraoperative	Bassini repair	Okoro et al. [10]
5	36	M	R	E	Incarceration	Intraoperative	Bassini repair	Kuyuma et al. [11]
6	NA	M	NA	NA		Intraoperative	Bladder repair and cystostomy	Tiryaki et al. [12]
7	4	M	NA	E		Intraoperative	Repair of bladder perforation and insertion of bladder catheter for 5 days	Aloi et al. [13]
8	3	M	B	E		Intraoperative		Shaw et al. [14]
9	12	M	R	E		Intraoperative	Repair of bladder perforation and insertion of bladder catheter for 5 days	Our patient
10	9	M	R	E		Intraoperative	Repair of bladder perforation and insertion of bladder catheter for 5 days	Our patient
11	9	M	NA	E	Wound infection and seeping from the wound	Postoperative		Shaw et al. [14]
12	3	M	B	E	Seeping from the right inguinal wound incision	Postoperative	Second bladder repair was performed at the age of 6 months. Sigmoidocoloplasty was performed at the age of 10 because of a small and contracted bladder.	Miyano et al. [15]
13	24	M	R	U	Peritonitis and ascites	Postoperative	Bladder repair	Ko et al. [16]
14	84	M	R	E	Seeping from the wound incision	Postoperative	Bladder repair and insertion of bladder catheter	Zajaczkowski [17]
15	18	M	R	E	Anuric with progressive abdominal distention	Postoperative	Laparotomy, closure of bladder almost completely resected with only base remaining	Chung et al. [18]
16	5	M	B	E		Postoperative elective cystourethrogram revealed bladder herniating into the right side day 2.	Premature (24 weeks), second operation Bassini repair	Dann et al. [19]
17	3	F	L	E	Abdominal distension, left groin bulge, oliguric	Postoperative ultrasound showed a large amount of intra-abdominal free fluid.	Premature, laparotomy, closure of bladder almost completely resected with only base remaining	Koot et al. [20]
18	4	M	R	E	Recurrent bulging at right scar after bilateral scrotal hernia repair	Postoperative	Premature (25 weeks), widened deep inguinal ring closure snugly (Bassini’s procedure?) at the age of 11 m	Tröbs et al. [21]
19	3	M	R	E	Recurrent bulging at right scar after bilateral scrotal hernia repair	Postoperative	Premature (26 weeks), widened deep inguinal ring closure snugly (Bassini’s procedure?) at the age of 5 m	Tröbs et al. [21]
20	3	M	L	U	Abdominal distension, vomiting	Postoperative cystogram demonstrated extravasation.	Bladder repair and cystostomy	Colodny [22].
21	18	M	R	E	Abdominal distension, anuria	Postoperative ultrasound showed intra-abdominal free fluid. Cystogram demonstrated extravasation and small capacity.	Bladder repair and cystostomy	Bakal et al. [23]
22	3	F	L	E	Abdominal distension	Postoperative	Laparotomy, closure of bladder almost completely resected with only base remaining	Wright [24]
23	NA	M	NA	NA	Seeping from inguinal wound incision	Postoperative	Suprapubic drainage and antibiotic treatment	Tiryaki et al. [12]
24	NA	M	NA	NA	Seeping from inguinal wound incision	Postoperative	Suprapubic drainage and antibiotic treatment	Tiryaki et al. [12]
25	6	M	NA	E	Peritonitis	Postoperative	Bladder repair	Aloi et al. [13]
26	3	M	NA	E	Retracted bladder with reflux and febrile urinary tract infection	Postoperative	Bladder augmentation and bilateral ureteral reimplantation	Aloi et al. [13]
27	1	M	R	E	Abdominal distention, seeping from the wound	Postoperative cystogram demonstrated extravasation.	Laparotomy, closure of bladder almost completely resected with only base remaining	Redman et al. [25]
28	1	M	B	E	Anuric	Postoperative cystogram demonstrated extravasation.	Bladder had been resected with both ureters. Bilateral ureteroileocecoplasty was performed.	Redman et al. [25]

## Data Availability

All data are included in the main manuscript.
